# Stimuli-Responsive Chitosan Hydrogels for Diabetic Wound Management: Comprehensive Review of Emerging Strategies

**DOI:** 10.3390/biomimetics10120807

**Published:** 2025-12-02

**Authors:** Selvam Sathiyavimal, Ezhaveni Sathiyamoorthi, Devaraj Bharathi, Perumal Karthiga

**Affiliations:** 1 Oral and Maxillofacial Surgery and Digital Implant Surgery Research Unit, Chulalongkorn University, Bangkok 10330, Thailand; 2 School of Chemical Engineering, Yeungnam University, 280 Daehak-Ro, Gyeongsan 38541, Republic of Korea; 22000138@ynu.ac.kr; 3 Department of Biotechnology, Shri Nehru Maha Vidyalaya College of Arts and Science, Coimbatore 641050, India

**Keywords:** chitosan hydrogels, stimuli-responsive, diabetic wounds, tissue regeneration

## Abstract

Diabetic wounds remain a major clinical challenge due to impaired angiogenesis, chronic inflammation, oxidative stress, and persistent infection, all of which delay tissue repair. Conventional dressings provide only passive protection and fail to modulate the wound microenvironment effectively. Chitosan (CS) is a naturally derived polysaccharide inspired by biological structures in crustaceans and fungi. It has emerged as a multifunctional biomimetic polymer with excellent biocompatibility, antimicrobial activity, and hemostatic properties. Recent advances in biomimetic materials science have enabled the development of stimuli-responsive CS hydrogels. These systems can sense physiological cues such as pH, temperature, glucose level, light, and reactive oxygen species (ROS). These smart systems emulate natural wound healing mechanisms and adapt to environmental changes. They release bioactive agents on demand and promote tissue homeostasis through controlled angiogenesis and collagen remodeling. This review discusses the biomimetic design rationale, crosslinking mechanism, and emerging strategies underlying single and dual-responsive hydrogel systems. It further emphasizes how nature-inspired structural and functional designs accelerate diabetic wound repair and outlines the current challenges and future prospects for translating these bioinspired intelligent hydrogels into clinical wound care applications.

## 1. Introduction

The increasing prevalence of diabetes worldwide has led to a rise in complications, particularly chronic wounds, which are often difficult to heal and highly susceptible to infection [1,2]. These non-healing wounds such as diabetic foot ulcers are a major cause of morbidity and may result in lower-limb amputation if not effectively treated [3]. The World Health Organization (WHO) and recent epidemiological studies project that diabetes could affect more than 700 million people globally by 2045. This rapid increase underscores the urgent need for innovative wound-care technologies [4]. Under healthy conditions, wound healing proceeds through a regulated sequence of biological events including clot formation, inflammation, tissue regeneration, and remodeling [5]. In diabetic patients, the healing process becomes impaired. This is due to metabolic abnormalities that interfere with normal tissue repair. Elevated blood glucose levels trigger oxidative stress, impair immune responses, inhibit angiogenesis, and promote bacterial colonization, all of which hinder tissue repair and prolong inflammation. This pathological state results in a wound environment that is difficult to resolve and often transitions into chronicity [6].

**Figure 1:**
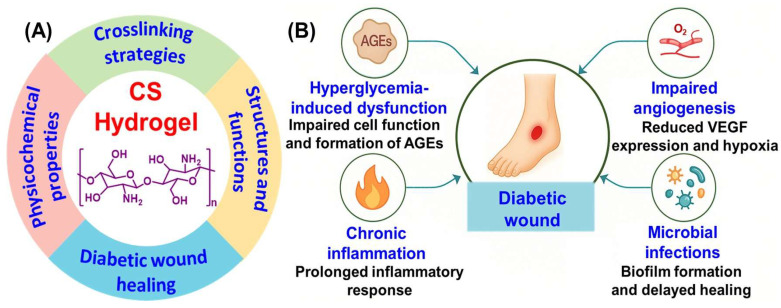
(**A**) Functional roles of CS hydrogel and (**B**) key complications in diabetic wound healing.

Conventional treatments such as gauze dressings provide only basic coverage and limited influence over the biochemical environment of the wound [7]. As a result, the medical field has turned its attention to functional materials like hydrogels that not only protect but also actively participate in the healing process. The term “gel” originates from the Latin word meaning snow or ice. Fundamentally, a gel is an elastic, semisolid system that exhibits both solid and liquid characteristics. It can retain its shape like a solid yet behaves as a viscous liquid under stress [8]. According to the United States Pharmacopeia (USP), gels are semisolid colloidal systems in which inorganic particles or organic macromolecules are uniformly dispersed within a liquid medium [9]. Hydrogels are particularly suited for wound management due to their high-water content, biocompatibility, and ability to support tissue regeneration by mimicking the extracellular matrix (ECM) [10]. Among the various biomaterials explored for hydrogel synthesis, chitosan (CS) a naturally derived polysaccharide has emerged as a leading candidate [11]. It possesses multiple properties such as antibacterial activity, biodegradability, and hemostasis, which are beneficial to wound healing [12]. CS can adhere to wound tissue, control microbial growth, and facilitate moisture retention, thereby creating a more favorable environment for repair [13]. Nonetheless, its native form has limitations such as poor mechanical resilience and pH-dependent solubility, which restrict its application in complex wound settings [14]. An illustration of the functional characteristic of CS is shown in Figure 1A.

The development of CS-based hydrogels is rooted in biomimetic principles, inspired by the adaptive and regenerative abilities of natural systems [15]. In nature, organisms exhibit self-healing, structural flexibility, and responsiveness to environmental cues-features that are now being replicated in synthetic hydrogel design. CS derived from chitin found in crustacean shells, embodies nature’s blueprint for strength, flexibility and biocompatibility [16]. Modern biomimetic material science applies these concepts to create hydrogels that mimic the ECM allowing cell attachment, nutrient transport, and biological signaling [17]. Stimuli-responsive CS hydrogel mimics the body’s natural feedback mechanisms, adjusting their structure or releasing therapeutic molecules in response to environmental signals such as pH, glucose or oxidative stress at the wound site.

Recent advances have addressed these issues through the development of modified CS hydrogels. Techniques such as nanoparticle incorporation, chemical crosslinking, and the design of injectable or stimuli-responsive systems have significantly improved their performance [18]. These innovations aim to enhance wound site delivery, promote tissue regeneration, and respond dynamically to the local wound environment [19]. This review provides a detailed analysis of the role of CS-based hydrogels in diabetic wound healing. It outlines the pathophysiological challenges posed by diabetic wounds, summarizes the key properties and functions of CS and evaluates the progress made in designing advanced hydrogel systems. Furthermore, it also discusses the translational potential and current limitations, paving the way for future research and clinical adoption of these next-generation wound care materials.

## 2. Pathophysiology of Diabetic Wounds

Chronic wounds in diabetic individuals arise from a complex interaction of systemic metabolic imbalances and local tissue impairments. These wounds often fail to progress through the normal stages of healing, coagulation, inflammation, proliferation, and tissue remodeling. As a result, they exhibit delayed closure and a higher risk of complications (Figure 1B) [5]. Elevated blood glucose levels significantly interfere with cellular activities essential for wound repair, as fibroblasts and keratinocytes show reduced proliferation and migration, while collagen synthesis is compromised [20]. Furthermore, chronic hyperglycemia promotes the accumulation of advanced glycation end products (AGEs), which disrupt ECM organization and trigger inflammatory responses via receptors for advanced glycation end products (RAGE)-mediated pathways. A hallmark feature of diabetic wounds is prolonged inflammation driven by pro-inflammatory macrophages (M1 phenotype) and elevated cytokines such as tumor necrosis factor (TNF)-α, Interleukin (IL)-1β, and IL-6. These factors inhibit tissue regeneration and promote ECM degradation through the overexpression of matrix metalloproteinases (MMPs). In addition, oxidative stress is exacerbated in diabetic wounds due to excess reactive oxygen species (ROS), which impair cell viability by damaging membranes, proteins, and DNA, while antioxidant defenses like glutathione are often depleted. Impaired vascularization further complicates wound healing in diabetics. Disrupted angiogenic signaling including reduced vascular endothelial growth factor (VEGF) and hypoxia inducible factor (HIF)-1α, resulting in localized hypoxia that hinders tissue repair and immune defense [20,21,22]. The compromised immune function and poor perfusion characteristic of diabetes also increase susceptibility to infection with pathogens like *Staphylococcus aureus* and *Pseudomonas aeruginosa* commonly forming biofilms that resist antibiotics and immune clearance [23,24]. Finally, matrix remodeling becomes dysregulated with MMPs upregulated and their inhibitors downregulated. This imbalance causes excessive ECM degradation and prevents scaffold formation for proper cell attachment and tissue regeneration. These challenges highlight the need for targeted therapies such as CS-based hydrogels [25].

## 3. Chemistry, Structure, and Sources of CS

**Figure 2:**
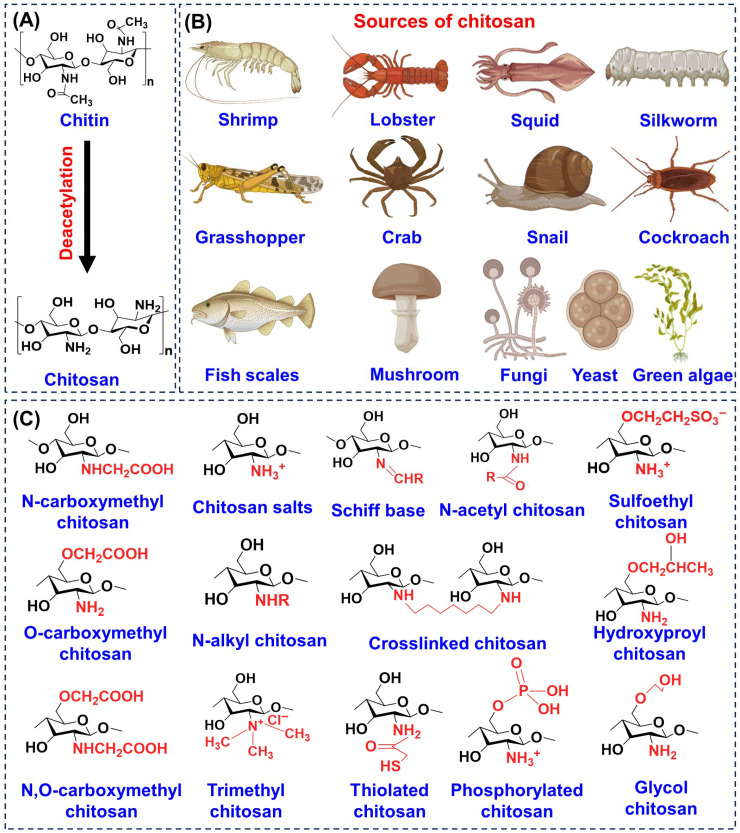
Overview of CS chemistry and sources. (**A**) Basic structure and key derivative of CS. (**B**) Natural sources including crustaceans, insects, mollusks, fungi, algae, and mushrooms. (**C**) Functionalized derivatives illustrating chemical modifications that enhance solubility, stability, and biomedical performance.

CS is a naturally derived linear polysaccharide that consists of repeating units of D-glucosamine and *N*-acetyl-D-glucosamine joined by β-(1→4) linkages [26], as illustrated in Figure 2A. Its chemical nature is largely determined by the abundance of reactive amino and hydroxyl groups along the backbone, which allow for hydrogen bonding, ion chelation, and a wide range of structural modifications [27]. Two important parameters that influence its properties are the degree of deacetylation (DD) and molecular weight [28]. Typically, the DD falls between 60 and 100%, and this strongly affects solubility, charge density, and bioactivity [29]. CS with a low molecular weight (below 100 kDa) is more soluble and readily bioavailable, making it especially useful for applications in wound repair, tissue regeneration, and drug delivery. Medium molecular weight material (approximately 190–310 kDa) offers a balance between solubility and viscosity, which is advantageous for controlled release systems, whereas high molecular weight CS (above 1000 kDa) forms stable gels and films with strong adhesive capacity, particularly suited for wound dressings and scaffolds that require extended performance [30,31,32].

The functional groups of CS, primarily the amino group at the C-2 position and hydroxyl groups at C-3 and C-6 are central to its biological functions [33]. They enable electrostatic interactions with negatively charged cell membranes, promote chemical derivatization, and contribute to its mucoadhesive behavior [34,35]. Beyond its native form, CS can be chemically modified to yield a wide range of derivatives, as shown in Figure 2C, including carboxymethyl, thiolated, phosphorylated, glycol, and trimethyl CS [36]. These modifications improve solubility at physiological pH, enhance stability, strengthen mucoadhesion, and expand antimicrobial or tissue-regenerative properties, thereby overcoming the limitations of unmodified CS. The natural sources of CS are diverse ranging from crustacean shells such as shrimp, crab, lobster to insects (silkworm, grasshopper, cockroach), mollusks (squid, snail), fungi, yeast, green algae, mushrooms, and even fish scales (Figure 2B). This wide availability highlights its renewable and abundant nature [37].

## 4. Advantages and Biomimetic Perspective of CS Hydrogels

Several natural and synthetic hydrogels including alginate, hyaluronic acid, gelatin, collagen, and synthetic polymers such as polyethylene glycol (PEG) and polyvinyl alcohol (PVA) have been widely applied in diabetic wound management due to their moisture-retention and biocompatibility [38,39,40,41,42,43]. However, these materials often lack intrinsic antimicrobial activity, hemostatic capability, or the ability to modulate inflammation, requiring additional modification or incorporation of active agents. In contrast, CS offers several inherent advantages including broad-spectrum antimicrobial activity, strong hemostatic action, mucoadhesive behavior. Its cationic structure also supports cell adhesion and promote re-epithelization [44,45,46]. Its reactive amino and hydroxyl groups also allow efficient chemical modification, enabling the development of stimuli-responsive, self-healing and multifunctional hydrogels. Beyond these material benefit, CS is biodegradable, sustainably source, and exhibits low toxicity, making it more versatile and multifunctional than many other hydrogel matrices [47].

These structural characteristics and derivatization strategies translate into important bioactivities relevant to promoting platelet aggregation for hemostasis, disrupting microbial membranes for antibacterial action, and enhancing granulation tissue formation, angiogenesis, and epithelialization for regeneration [48]. Clinical studies further confirmed these benefits, demonstrating that CS-based hydrogels accelerate wound closure and improve overall healing outcomes [49,50]. From a biomimetic perspective, CS and its derivatives exemplify a natural model for material design. Their cationic nature and hydrated network mimic the ECM, supporting cell adhesion, migration and nutrient diffusion, where biological environments respond dynamically to pH, or oxidative stress. These properties collectively position CS based hydrogels as bioinspired and adaptive materials that emulate the body’s intrinsic healing, aligning closely with the principles of biomimetics.

## 5. Mechanism of CS Based Responsive Formation

CS-based responsive hydrogels are typically fabricated through physical or chemical crosslinking approaches. The choice of method largely determines their structural stability, responsiveness, and biomedical applicability [51,52]. These strategies are designed to impart stimuli-responsiveness to the hydrogel. As a result, the material can undergo reversible swelling, degradation, or sol–gel transitions in response to triggers such as pH, temperature, redox potential, enzymatic activity, or even multiple combined stimuli [53]. These crosslinking strategies draw inspiration from natural self-assembly and adhesion processes. They reflect the core biomimetic principle of replicating nature’s hierarchical and adaptive material organization. The following sections provide detailed approaches.

### 5.1. Physical Crosslinking Methods

Physical crosslinking relies on non-covalent interactions such as ionic bonds, hydrogen bonding, hydrophobic associations, or on physical processing techniques like freeze–thaw cycles and host-guest inclusion [54]. These methods do not require potentially toxic chemical reagents, making them particularly attractive for wound healing applications. Hydrogels formed by physical crosslinking are typically biocompatible, reversible, and often injectable, though they usually exhibit lower mechanical strength compared with chemically crosslinked counterparts [55,56].

#### 5.1.1. Ionic/Electrostatic Interaction

**Figure 3:**
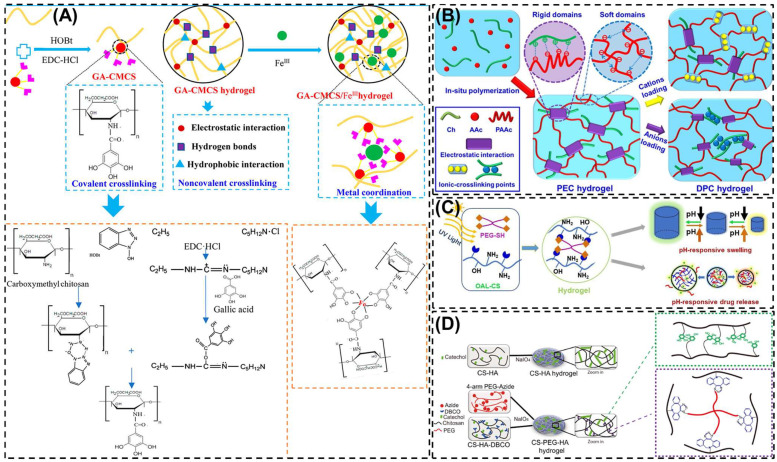
Schematic representation of the various formation mechanism of CS based hydrogels. (**A**) Schematic diagram for the preparation of GA-CMCS hydrogel and GA-CMCS/Fe ^III^ hydrogel. (**B**) Illustration of the preparation of PEC and DPC hydrogels (**C**) Formation of C_6_ O-allyl-CS hydrogel by UV-triggered thiol-ene click chemistry. (**D**) Formation of CS-HA and CS-PEG-HA hydrogel. Reprinted with permission from Yang et al. [65] (Copyright, 2019, American Chemical Society (ACS)), Cao et al. [57] (Copyright, 2020, Elsevier), Ding et al. [89] (Copyright, 2021, Elsevier) and Li et al. [90] (Copyright, 2022, Elsevier).

In acidic media, CS possesses protonated amino groups that readily interact with multivalent anions such as sodium tripolyphosphate (STP), citrate, sulfate or phosphate [35]. These electrostatic interactions facilitate rapid hydrogel formation under mild conditions. The method is straightforward, non-toxic, and compatible with sensitive bioactive molecules. Cao et al. [57] developed polyelectrolyte complex (PEC) hydrogels through the in situ polymerization of acrylic acid monomers within a CS solution (Figure 3B). Subsequently, various cations (Na^+^, Mg^2+^, and Al^3+^) and anions were incorporated into the PEC network to establish strong electrostatic interactions with the polymer chains, leading to the formation of double polyelectrolyte complex (DPC) hydrogels. The incorporation of these ions played a critical role in determining the mechanical performance of the resulting hydrogels. The tensile strengths of the ion-modified DPC hydrogels (2.36, 12.59, 65.1, and 2.80 MPa, respectively) were markedly higher than that of the blank control (0.29 MPa). Notably, the DPC–Ca hydrogels exhibited distinct ionic conductivity and exceptional anti-freezing behavior, remaining unfrozen even at −20 °C. This DPC–Ca strategy provides a promising route for the design of CD-based hydrogels with enhanced mechanical strength, conductivity, and temperature tolerance. Wang et al. [58] designed an injectable CS-based hydrogel by utilizing CS as the primary polymeric matrix. The amino groups on the CS backbone enabled the conjugation of catechol moieties, mimicking mussel-inspired adhesive proteins, through electrostatic interactions and cooperative reactions with the sulfonic acid groups of the thermosensitive monomer N-isopropylacrylamide. This innovative formulation produced a biodegradable, biocompatible, and temperature-responsive hydrogel with remarkable tissue adhesion and stability, making it highly promising for biomedical and wound-healing applications. Their fast swelling and controlled release properties make them particularly useful for drug delivery applications. However, such hydrogels often suffer from limited mechanical integrity and tend to disintegrate quickly under physiological conditions.

#### 5.1.2. Hydrogen Bonding

Hydrogen bonding plays a significant role in stabilizing CS hydrogels, either through interactions between the hydroxyl and amino groups of CS itself or in blends with other polymers such as polyvinyl alcohol (PVA) and poly (ethylene glycol) (PEG) [59]. These reversible interactions contribute to pH- or temperature-responsive behavior. For instance, hydrogen bonding in CS–PVA hydrogels enhances elasticity and swelling control. In a study, Yan et al. [60] employed agar and CS to demonstrate the fabrication of a double network hydrogel via strong H-bonding between the -OH group of agars and the NH_3_^+^ group CS. Another study by Han and coworkers reported the fabrication of hydrogel reinforced cellulose nanocrystals through electrostatic and hydrogen bonding interaction between xanthan solution and CS [61]. These studies revealed that hydrogels can be fabricated via simple hydrogen bond formation and can be made stimuli-responsive. Nonetheless, hydrogen-bonded networks are often sensitive to environmental fluctuations and may lack long-term stability [62].

#### 5.1.3. Hydrophobic Interaction

Hydrophobic modification of CS involving grafting with alkyl chains, cholesterol, or aromatic groups, induces self-association in aqueous environments. These hydrophobic domains aggregate, forming a 3D-network. In general, hydrogels can be synthesized by utilizing both techniques and the resultant gels can be tailored to achieve sol-to-gel transition under specific conditions. Hydrogels can also be structured by regulating hydrophobic interactions, which are achieved mainly through two fabrication routes. In the first route, micellar structures act as dynamic crosslinkers, where the hydrophobic cores not only entrap poorly water-soluble drugs but also connect polymer chains to form a stable network. This technique effectively enhances the drug-loading efficiency, distribution uniformity, and overall stability of the hydrogel [63]. The second route involves introducing hydrophobic segments such as long alkyl, steroidal, or aromatic groups onto otherwise hydrophilic polymer backbones. This modification promotes spontaneous self-association in aqueous media, leading to a three-dimensional network held together by hydrophobic domains [64]. In addition, hydrophobic interactions can be triggered or strengthened through two external signals: thermal modulation, utilizing phase transition behavior such as lower critical solution temperature (LCST) or upper critical solution temperature (UCST), and ultrasonic treatment, which facilitates molecular aggregation and network formation [52]. Together, these approaches allow the development of robust, stimuli-responsive hydrogels with improved capacity to incorporate and deliver hydrophobic bioactive molecules. Hydrogels prepared through this mechanism frequently display temperature- or pH-dependent responsiveness, since hydrophobic interactions are strengthened under physiological conditions. A major advantage is their capacity to encapsulate and protect hydrophobic drugs and proteins, although achieving reproducible and structurally uniform gels remains a challenge.

#### 5.1.4. Metal-Chelating

The metal-chelating method utilizes the abundant amino and hydroxyl groups of CS to coordinate with multivalent metal ions, forming stable crosslinked hydrogel networks. Transition metals such as Fe^3+^, Cu^2+^, Zn^2+^ or Ca^2+^ interact electrostatically or through coordination bonds with the functional groups of CS, resulting in improved mechanical strength, structural integrity and antibacterial activity. The reversible nature of metal-ligand coordination also imparts self-healing and stimuli-responsive properties as the crosslinkers can dissociate and reform under changes in pH or ionic strength. For instance, Yang et al. [65] prepared gallic acid-modified carboxymethyl chitosan/ferric ion (GA-CMCS/Fe ^III^) composite hydrogels using non-covalent interaction and metal coordination (Figure 3A). GA-CMCS/Fe^3+^ composite hydrogels showed good compressive strength and reasonable mechanical strength, providing a new opportunity to construct physical multi-crosslinking systems with excellent mechanical strength. Metal-chelated CS hydrogel offers simple fabrication, improved mechanical strength, and pH responsive behavior. However, excessive metal-ion release during degradation may reduce long-term stability under fluctuating pH or ionic environments.

#### 5.1.5. Freeze–Thaw Method

Freeze–thaw is one of the physical cross-linking techniques, which is primarily accomplished by single or repeated cycles. When CS is blended with PVA and subjected to repeated freeze–thaw cycles, crystallite regions form that act as physical junction points, stabilizing the hydrogel network [66]. This approach eliminates the need for chemical crosslinkers and produces non-toxic hydrogels with enhanced elasticity and toughness [67]. Such hydrogels have shown promise in wound dressings and biomedical scaffolds [68]. However, multiple cycles are usually required to obtain adequate stability and their swelling properties may still be limited compared to chemically crosslinked systems.

#### 5.1.6. Host-Guest Interaction

This host-guest mechanism generates supramolecular hydrogels with reversible gelation and tunable responsiveness [69]. Cyclodextrins (CDs) are widely employed as host molecules to form inclusion complexes with hydrophobic guest moieties attached to CS [70]. For example, CS modified with adamantane (guest) can interact with β-cyclodextrin (host) to yield self-assembled networks [71]. These hydrogels are particularly valuable in drug delivery and tissue engineering, where controlled responsiveness and reversible crosslinking are advantageous. Host–guest interaction hydrogels can also be engineered to respond to external stimuli such as temperature, pH, or light, making them highly suitable for controlled and targeted drug delivery applications. Their main limitation lies in the relatively weak binding affinities of host–guest pairs under physiological conditions, which may compromise stability.

### 5.2. Chemical Crosslinking Methods

Chemical crosslinking produces covalent bonds between or within polymer chains, resulting in robust, permanent hydrogel networks. These networks offer superior mechanical strength, tunable degradation, and highly controlled swelling behavior [72]. Unlike physically crosslinked hydrogels, these systems are more resistant to dissolution under physiological conditions, making them particularly attractive for load-bearing wound healing applications. However, the cytotoxicity of some crosslinkers and the need for careful purification remain important considerations.

#### 5.2.1. Free Radical Polymerization

Free radical polymerization is widely used to graft monomers such as acrylic acid, acrylamide, or N-isopropylacrylamide onto the backbone [68]. This approach generates hydrogels with enhanced stimuli-responsiveness, including pH-sensitivity (via acrylic acid) and thermo-responsiveness (via poly (N-isopropylacrylamide) PNIPAM) [73]. In a typical free-radical polymerization process, four fundamental stages occur: initiation, propagation, chain transfer, and termination. During initiation, reactive radical species are generated by external triggers such as ultraviolet or visible light, heat, or redox reactions [74]. These radicals attack monomer units, creating active growing centers. In the propagation stage, these centers continuously add monomer molecules, allowing the polymer chains to extend. Eventually, the reaction is terminated when two radical chains combine or a chain transfer reaction takes place, producing a stable crosslinked polymeric network that forms the hydrogel structure [75]. These grafted networks provide high versatility and tunability of mechanical and physicochemical properties. Nevertheless, residual initiators or unreacted monomers must be carefully removed to ensure safety for biomedical use.

#### 5.2.2. Enzymatic Crosslinking

Enzymatic methods offer a greener alternative, where enzymes such as horseradish peroxidase (HRP), tyrosinases, peroxidases, phosphopantetheinyl transferase, transglutaminase catalyze specific reactions in derivatives [76,77]. For example, HRP can crosslink gallic acid onto the CS backbone [78], while transglutaminase are the members of the thiol group of enzyme that catalyzes amide bonds between carboxylic and amide/amino groups in the presence of calcium ions as cofactors [79]. These processes yield biocompatible hydrogels under physiological conditions with excellent cytocompatibility, spatiotemporal control, and suitability for cell encapsulation. Limitations include higher cost and sensitivity to enzyme activity and storage conditions.

#### 5.2.3. Photo-Crosslinking

Photo-crosslinking has emerged as an effective approach for developing CS-based hydrogels suitable for a wide range of biomedical uses, including drug delivery, tissue repair, and wound healing. The process involves incorporating light-responsive groups or photoinitiators that can absorb energy from ultraviolet or visible light and produce reactive species capable of linking polymer chains into a stable three-dimensional network [76]. To ensure biological safety, non-toxic and biocompatible initiators such as riboflavin, eosin Y, or camphorquinone are typically employed [62,80,81]. Hydrogels produced by photo-crosslinking generally exhibit good cytocompatibility, improved mechanical behavior, and efficient cell encapsulation, making them promising candidates for tissue-engineered scaffolds [82]. Additionally, this technique provides spatiotemporal control over gelation, allowing hydrogel formation to occur in situ and without direct physical manipulation, which is particularly beneficial for irregular or deep wound sites.

#### 5.2.4. Click Chemistry

Click chemistry represents a group of highly efficient and selective reactions designed to covalently attach functional molecules to specific reactive sites on another compound [83]. These reactions generally occur under mild, catalyst-friendly, and high-yielding conditions, and they are largely unaffected by the presence of other species in the reaction mixture [84,85]. Because of these advantages, click chemistry has become a preferred approach for constructing hydrogel networks with precise structural control. In hydrogel crosslinking, click-based strategies are commonly categorized into five principal reaction types: the Huisgen cycloaddition, thiol-ene coupling, Diels-Alder cycloaddition, Michael addition, and Schiff-base condensation [86,87,88]. These reactions are highly efficient, selective, and bio-orthogonal, proceeding under mild conditions with minimal byproducts. For example, in the modified C_6_ O-allyl (OAL)-CS system, the thiol-containing crosslinkers generates thiol radicals under UV irradiation, which undergo a thiol-ene reaction with allyl groups on OAL-CS to rapidly form hydrogel, as shown in Figure 3C [89]. The solubility of the UV-triggered thiol-ene hydrogels varies with the pH of the surrounding medium, enabling their use as a pH-responsive slow drug release system. The resulting networks offer excellent reproducibility and structural control, making them well suited for drug delivery, biosensing, and regenerative medicine. The main challenge lies in the need to pre-functionalize CS with reactive moieties, which adds complexity to the preparation process.

#### 5.2.5. Graft Copolymerization Techniques

Graft copolymerization is an effective strategy to modify CS and enhance its physiochemical and biological properties for hydrogel fabrication. In this approach, synthetic or natural monomers are covalently grafted onto the CS backbone through reactive amino or hydroxyl groups, which improve solubility, mechanical strength, stability and stimuli-responsiveness. Li et al. [90] successfully developed CS-polyethylene glycol-hydro caffeic acid composite hydrogels, in which catechol groups were grafted onto CS chains via 1-Ethyl-3-(3-dimethylaminopropyl) carbodiimide (EDC)-mediated coupling chemistry, as illustrated in Figure 3D. The resulting CS-PEG-HA hydrogels exhibited excellent self-healing ability and effectively promoted angiogenesis and epidermal regeneration. Such self-healing, chemically crosslinked hydrogels broaden the potential of CS-based materials for advanced biomedical and regenerative applications.

### 5.3. Self-Assembly Methods

Self-assembly represents a unique strategy for the preparation of CS hydrogels, relying on the intrinsic amphiphilicity and charge of CS or its derivatives to spontaneously organize into 3D networks under aqueous conditions [91]. These methods do not require chemical crosslinkers, making them attractive for injectable and in situ gelling systems. However, the resulting hydrogels often show relatively weaker mechanical properties compared with covalently crosslinked systems.

One common approach is polyelectrolyte complexation, where positively charged interacts with negatively charged biopolymers such as alginate, carrageenan, hyaluronic acid, or DNA [92]. The electrostatic attraction between these oppositely charged chains leads to hydrogel formation with high biocompatibility and biodegradability. Such systems are particularly well suited for wound dressings, gene delivery, and tissue regeneration. Another important approach involves amphiphilic derivatives, where hydrophobic chains are grafted onto CS backbones. In aqueous environments, these derivatives undergo micellization and self-aggregation, forming hydrogel networks that can respond to pH, temperature, and ionic strength. These hydrogels are especially useful for the encapsulation and controlled release of hydrophobic drugs or proteins [93]. Table 1 represent the classification of CS-based hydrogel-based on crosslinking mechanisms.

**Table 1:** Classification of CS-based hydrogels according to crosslinking mechanism, representative crosslinkers, advantage, limitation and major biomedical applications.

Category	Method	Mechanism/Crosslinker	Advantages	Limitations	Applications	References
Physical crosslinking	Ionic/electrostatic	Electrostatic interaction between protonated and multivalent anions (TPP, citrate, sulfate, phosphate)	Simple, non-toxic, mild conditions and biocompatible	Poor mechanical strength and unstable in physiological media.	Drug delivery and controlled release	[94,95,96]
	Hydrogen bonding	Intermolecular hydrogen bonds (–OH, –NH_2_ groups) or blending with PVA and PEG.	Reversible, responsive to pH/temperature and enhances elasticity	Sensitive to environment and weak long-term stability	Swelling control and wound dressing	[97,98,99]
	Hydrophobic interaction	Grafted alkyl, cholesterol and aromatic moieties induce aggregation	Encapsulation of hydrophobic drugs and pH/temperature responsiveness	Poor reproducibility and structural heterogeneity	Protein/drug delivery and responsive carriers	[100,101]
	Freeze–thaw	Repeated freeze–thaw cycles with PVA form crystallite junctions	Non-toxic, reinforced elasticity and toughness	Multiple cycles required and moderate swelling	Wound dressings and biomedical scaffolds	[102,103,104]
	Host-guest inclusion	Cyclodextrin (host) + hydrophobic CS moiety (guest)	Reversible, tunable and injectable	Weak host–guest binding under physiological conditions	Drug delivery and tissue engineering	[105,106,107]
Chemical crosslinking	Free radical polymerization	Grafting monomers (acrylic acid, N-isopropylacrylamide (NIPAM)) onto CS	Strong, versatile and stimuli-responsive networks	Toxic residual initiators and complex purification	Smart drug release and responsive gels	[108,109]
	Enzymatic	HRP or transglutaminase catalyzed crosslinking.	Biocompatible, mild and spatiotemporal control	High cost and enzyme sensitivity	Injectable gels and tissue regeneration	[78,110]
	Photo-crosslinking	UV/visible light crosslinking of methacrylated/cinnamate CS	Precise spatial/temporal control and rapid gelation.	UV cytotoxicity and photo initiator concerns	3D bioprinting and localized delivery.	[111,112,113]
	Click chemistry	Azide-alkyne or thiol-ene reactions	High specificity, reproducibility and minimal byproducts	Requires functionalization, and added complexity	Drug delivery, biosensing, and regenerative medicine	[114,115]
Self-assembly	Polyelectrolyte complexation	Electrostatic interaction with alginate, carrageenan, HA, and DNA	Mild, biocompatible, and biodegradable	Weak stability, less mechanical strength	Gene delivery, wound healing	[116,117]
	Amphiphilic derivatives	Hydrophobic modification (alkyl, cholesterol, PNIPAM)-micellization and aggregation	Multi-stimuli responsive and encapsulates hydrophobic drugs	Structural inconsistency with weak strength	Drug/protein delivery and smart carriers	[118,119,120]

## 6. Stimuli-Responsiveness in Diabetic Wound Healing

**Figure 4:**
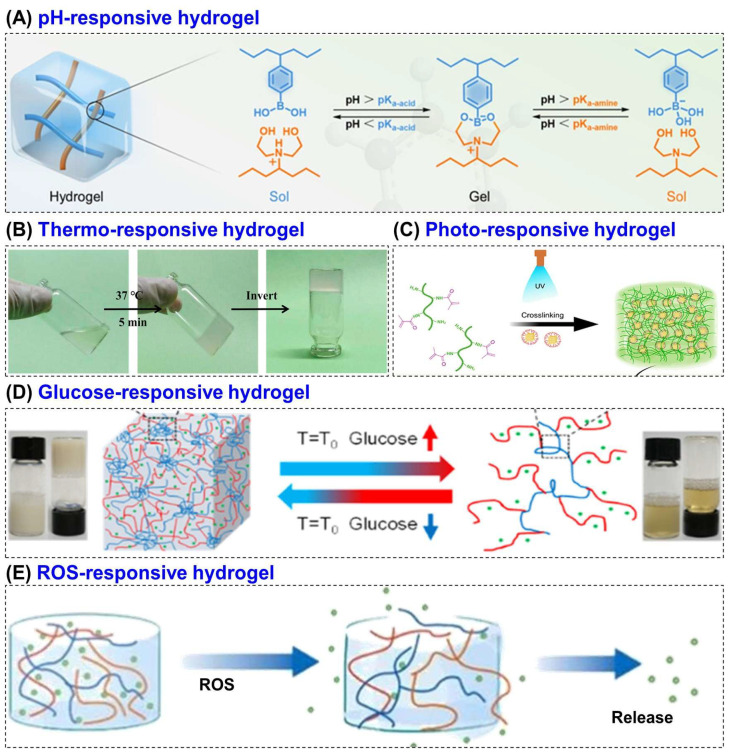
Schematic representation of various stimuli-responsive hydrogels, including (**A**) pH, (**B**) thermo, (**C**) photo, (**D**) glucose, and (**E**) ROS-responsive systems. Reprinted with permission from Kang et al. [128] (Copyright, 2023, ACS), Liu et al. [129] (copyright, 2022, MDPI), Kurian et al. [130] (copyright, 2023, Elsevier), Hu et al. [131] (Copyright, 2021, ACS) and Zhao et al. [132] (copyright, 2024, Elsevier).

Stimuli-responsive hydrogels are a class of smart polymeric materials that can sense and react to environmental changes by altering their physical or chemical properties [121]. These hydrogels respond to specific stimuli such as pH, temperature, light, glucose concentration, ROS or enzymatic activity leading to controlled swelling, degradation or therapeutic release [122,123]. Depending on the nature of the stimulus, they are categorized into several types such as pH, thermosensitive, photo, glucose and ROS-responsive hydrogel [124,125] (Figure 4). Among various polymers used to fabricate such systems, CS has gained significant attention due to its unique chemical functionality, physiological compatibility, enzymatic degradability, and inherent bioactivity. The presence of –OH and –NH_2_ groups in its molecular structure allows facile chemical modification, enabling the incorporation of responsive moieties that make the hydrogel sensitive to physiological stimuli [126].

In the context of diabetic wound healing, where the wound environment is often acidic, infected, and rich in glucose or ROS, these smart hydrogels offer multiple therapeutic benefits [18]. They can deliver drugs, growth factors, or signaling molecules in response to local stimuli while maintaining a moist and antimicrobial microenvironment. Additionally, they promote angiogenesis, collagen deposition, and overall tissue regeneration [127]. A comparative analysis of single and dual-stimuli-responsive CS-based hydrogel including their triggering mechanisms, major therapeutic advantages and current limitation are summarized in Table 2. The following sections provide a detailed overview of each type of stimuli-responsive CS hydrogel and their specific mechanism contributing to diabetic wound healing.

**Table 2:** Comparative analysis of single and dual-stimuli responsive CS-based hydrogel for diabetic wound healing.

Hydrogel Type	Stimulus/Trigger	Example Representative Composition	Key Advantage	Limitations	References
pH- responsive	Acidic microenvironment in infected wounds	Quaternary ammonium CS + oxidized dextran-dopamine (OD-DA) + silver nanoparticles (AgNPs)/deferoxamine (DFO)	Enables acid-triggered antibacterial and angiogenic release, good adhesion and self-healing	Limited mechanical strength, and possible instability under neutral pH	[133]
Thermo- responsive	Body temperature	CS + beta-glycerophosphate + Cu/Mg-MOF nano enzyme	Injectable, in situ gelation, modulates inflammation and promotes angiogenesis	Slow gelation and temperature-sensitive stability	[134]
Photo- responsive	Near-infrared (NIR) light	Carboxymethyl CS + gelatin + polydopamine-coated ZIF-8 NPs	On demand antibacterial activity via mild photothermal effect and enhances angiogenesis	Require external light sources, limited source and limited tissue penetration	[135]
Glucose- responsive	Elevated glucose concentration	CS + hyaluronic acid + L-arginine + glucose oxidase (GOx)	Self-regulated NO generation, improved angiogenesis and collagen synthesis	Complex fabrication and limited long term stability	[136]
ROS- responsive	High oxidative stress	Quaternized CS + metal–organic framework (MOF) enzymes	Scavenges ROS, restores redox balance, promotes oxygen generation and tissue repair	Costly nanozyme preparation and unclear biodegradation profile	[137]
Dual- responsive	pH/ROS or ROS/glucose	Quaternized CS + phenylboronic acid + catechol derivatives	Synergistic response to multiple stimuli and enhanced control over drug release	Complicated synthesis and reproducibility issues	[138,139]

### 6.1. pH-Responsive CS Hydrogel

During the wound healing process, the pH level of the wound site serves as an important indicator of healing status. Normal wounds generally exhibit a neutral or slightly alkaline pH (around 7–8), which promotes cellular activity and tissue regeneration. In contrast, diabetic wounds often shift toward an acidic environment (pH-6.5) as a result of bacterial infection, inflammation, and limited oxygen supply, which delays the healing process [140,141,142]. pH-responsive CS hydrogels are designed to detect this acidic condition and release therapeutic agents such as antibacterial compounds or growth-promoting molecules on demand [143]. Their sensitivity originates from the ionizable amino groups (–NH_2_) in the CS structure, which can gain or lose protons depending on pH fluctuations. These reversible interactions enable the hydrogel to swell, contract, or degrade as needed. This allows precise drug release, localized antibacterial action, and restoration of a favorable wound environment that supports tissue repair [144,145].

**Figure 5:**
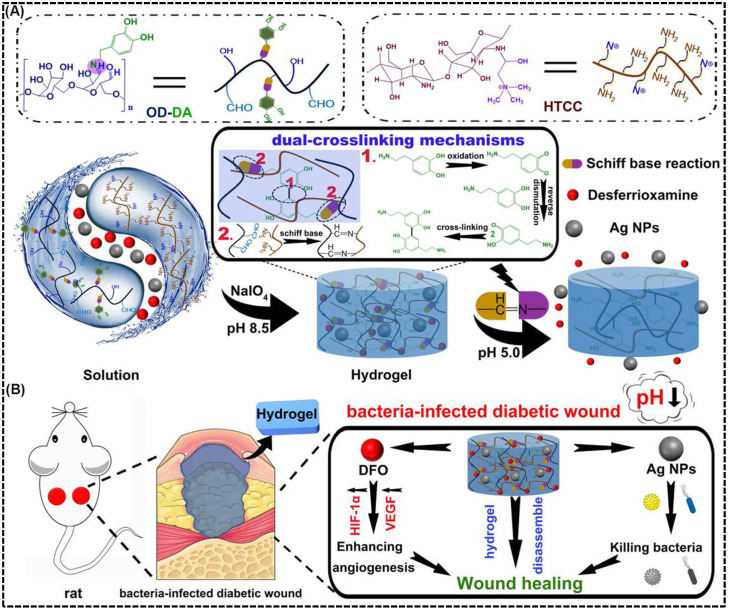
pH-responsive antibacterial and angiogenic CS-based hydrogel for diabetic wound healing. (**A**) Schematic illustration of the preparation and dual-crosslinking mechanism of the mussel-inspired hydrogel composed of OD-DA and HTCC. The hydrogel is formed through dynamic Schiff base reactions and coordination with AgNPs. This structure enables pH-triggered degradation and drug release under acidic wound conditions. (**B**) Illustration of the CS-based hydrogel applied to a bacteria-infected diabetic wound, showing pH-triggered disassembly that releases AgNPs and DFO. The release AgNPs provide antibacterial action, while DFO promote angiogenesis through HIF-1α/VEGF signaling to enhance wound healing. Reprinted with permission from Hu et al. [133] (copyright, 2021, Elsevier).

Several studies have been carried out to investigate the therapeutic potential of pH-responsive CS based hydrogels for diabetic wound healing applications. For example, Hu et al. [133] designed a dual-crosslinked, mussel-inspired pH-responsive CS hydrogel by coupling quaternary ammonium CS (HTCC) with oxidized dextran–dopamine (OD–DA) through Schiff base linkages and catechol–catechol interactions. This approach resulted in a self-healing, injectable, and adhesive hydrogel matrix (Figure 5A). This dual-crosslinking strategy provided the hydrogel with enhanced mechanical strength, structural stability, and dynamic responsiveness. To achieve multifunctionality, AgNPs and the angiogenic agent DFO were incorporated as active therapeutic components. Under the acidic conditions typical of infected diabetic wounds, the imine bonds within the hydrogel were cleaved, resulting in pH-triggered release of AgNPs for potent antibacterial action and DFO for angiogenic stimulation via HIF-1α and VEGF pathway activation (Figure 5B). Furthermore, in vivo studies in diabetic rat models demonstrated significantly accelerated wound closure (~79% within 7 days), enhanced collagen deposition, reduced macrophage infiltration, and robust neovascularization compared with control groups. This hydrogel’s acid-responsive degradation, self-repairing behavior, and moist, adhesive surface synergistically contributed to effective bacterial elimination and rapid tissue regeneration, making it a promising therapeutic platform for treating chronic and infected diabetic wounds.

Another study by Yang et al. [146] developed a pH-responsive hydrogel composed of tannic acid, quaternized carboxymethyl CS, oxidized sodium alginate, and carbon quantum dots for diabetic wound healing and real-time pH monitoring. The dynamic Schiff-base bonds between quaternized carboxymethyl CS (QCMCS) and oxidized sodium alginate (OSA) endowed the system with clear pH sensitivity, enabling controlled drug release and structural self-healing under acidic wound conditions. In diabetic mice, this pH-responsive CS-based hydrogel achieved about 97% wound closure within 12 days, enhanced angiogenesis, and offered simultaneous therapeutic and diagnostic benefits for advanced diabetic wound management. An injectable, self-healing, pH-responsive CS hydrogel for sustained insulin delivery in diabetic wound healing was developed by Li et al. [147] from N-carboxyethyl CS, hyaluronic acid–aldehyde, and adipic acid dihydrazide. The developed hydrogel exhibited acid-triggered degradation and controlled insulin release under wound-like pH. It also maintained insulin bioactivity, promoted cell migration, and achieved accelerated wound closure, angiogenesis, and collagen deposition in diabetic rats. These results suggest that pH-responsive CS hydrogels are simple, biocompatible systems that effectively release therapeutic agents in acidic wound environments. They show strong antibacterial and angiogenic activity but depend heavily on wound pH. Their main limitation are weak mechanical stability and short-term responsiveness, which restrict sustained use.

### 6.2. Thermo-Responsive CS Hydrogel

Temperature is critical in human physiology and can be conveniently controlled in both in vitro and in vivo conditions [148]. Thus, temperature-responsive hydrogel has attracted much attention in the past few decades. Thermo-responsive hydrogels can reversibly switch between sol and gel states with temperature, typically forming gels at body temperature (~37 °C) [149]. This property enables injectable and in situ-forming wound dressings that conform to irregular diabetic wounds and provide sustained drug release [150]. The phase transition of thermo-responsive hydrogels depends on their critical solution temperature, which can be classified as either lower critical solution temperature (LCST) or upper critical solution temperature (UCST). Hydrogels with a LCST undergo gelation when the temperature exceeds this threshold, whereas those with a UCST form gels when the temperature falls below it [151]. The temperature sensitivity is often achieved by blending CS with β-glycerophosphate (β-GP), poly (N-isopropylacrylamide) (PNIPAM), or Pluronic F127, allowing rapid gelation under physiological conditions [152,153].

**Figure 6:**
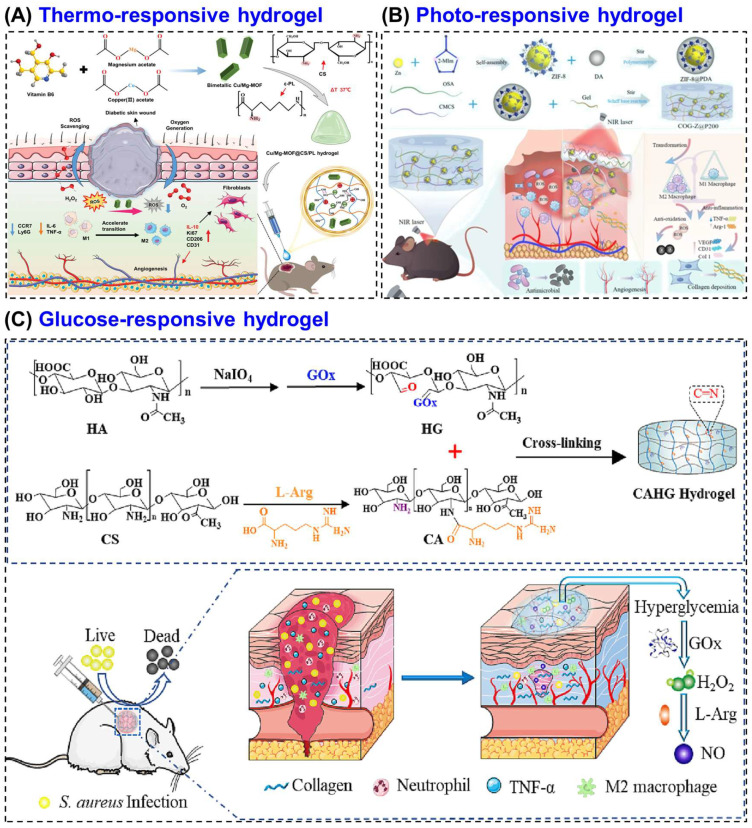
CS-based hydrogel for diabetic wound healing. (**A**) Schematic illustration of thermosensitive CS-based hydrogel incorporating Cu/Mg-MOF nano-enzymes to promote diabetic wound healing. (**B**) An illustration of the photo-responsive multifunctional hydrogel (COG-Z@P200) for methicillin resistant *Staphylococcus aureus* (MRSA)-infected diabetic wound healing. (**C**) A glucose responsive hydrogel prepared from CS, hyaluronic acid, and L-arginine (L-Arg) to promotes angiogenesis, collagen synthesis, and antibacterial defense in diabetic wounds. Reprinted with permission from Zhang et al. [134] (copyright, 2025, Wiley-VCH GmbH), Gao et al. [135] (copyright, 2025, BMC), and Zhou et al. [136] (copyright, 2023, Elsevier).

For examples, a thermosensitive CS hydrogel was prepared by grafting NIPAM onto carboxymethyl CS and reinforcing it with graphene [154]. The hydrogel showed gelation near body temperature (~34 °C), strong antibacterial activity, and excellent biocompatibility, making it a promising temperature-responsive system for drug delivery and wound healing. Zhang et al. [134] developed a thermosensitive CS-based hydrogel incorporating copper–magnesium bimetallic MOF (Cu/Mg-MOF) nano-enzymes to promote diabetic wound healing by regulating the wound microenvironment. The hydrogel, composed of CS and ε-polyline (PL) with β-glycerophosphate as the thermal gelling agent, that exhibited excellent injectability, temperature sensitivity, and biocompatibility, forming a stable gel at physiological temperature. They also demonstrated in vitro studies, which showed that the hydrogel effectively protected fibroblasts from oxidative stress, stimulated their migration, and modulated macrophage polarization by suppressing pro-inflammatory (M1) activity and enhancing anti-inflammatory (M2) behavior. In vivo diabetic mouse experiments revealed accelerated wound closure (~90.6% within 14 days) along with enhanced collagen formation, angiogenesis, and re-epithelialization (Figure 6A). These thermos-responsive hydrogels maintain a moist, antibacterial environment, while incorporated bioactive agents such as VEGF or insulin enhance angiogenesis, collagen synthesis, and tissue repair [155]. Thermos-responsive hydrogel offers good injectability and form stable gels at body temperature, enabling localized therapy. They promote angiogenesis and tissue repair but have slow gelation kinetics and limited temperature stability. Compared to other stimuli, they are less specific to diabetic biochemical cues.

### 6.3. Photo-Responsive CS Hydrogel

Photo-responsive CS hydrogels are a class of light-activated biomaterials that can transform light energy into therapeutic effects such as localized heating, drug release, or ROS generation [156]. These hydrogels are typically constructed by embedding photoactive agents for example, gold nanoparticles (AuNPs) [157], carbon quantum dots (CQDs) [158], or photosensitizers into a CS-based matrix. Upon exposure to visible or near-infrared (NIR) light, these photoactive components trigger controlled physicochemical changes within the hydrogel, allowing on-demand antibacterial, anti-inflammatory, and regenerative actions [159,160].

In normal healing, the tissue environment naturally supports inflammation resolution, collagen remodeling, and angiogenesis [161]. Here, photo-responsive CS hydrogels primarily act as antibacterial and regenerative enhancers, accelerating closure by sterilizing the wound surface, stimulating fibroblast proliferation, and maintaining a moist healing environment [162]. The mild photothermal or photodynamic effects produced under light exposure can promote microcirculation, thereby speeding up re-epithelialization and granulation tissue formation without damaging healthy cells. For example, Wang et al. [163] compounded that the dopamine-assisted exfoliated molybdenum disulfide into lipoic acid modified CS (LAMC-MoS_2_@PDA) hydrogel promotes wound healing through a photothermal antibacterial and tissue-regenerative mechanism. Under 808 nm NIR irradiation, the MoS_2_ nanosheets convert light into mild localized heat, effectively eliminating bacteria and reducing inflammation. Simultaneously, the CS matrix supports cell proliferation, collagen synthesis, and angiogenesis, creating a favorable microenvironment that accelerates normal wound closure and tissue regeneration.

In contrast, diabetic wounds are characterized by persistent infection, excessive oxidative stress, reduced oxygen supply, and impaired angiogenesis [164]. In such a pathological microenvironment, photo-responsive CS hydrogels provide a more complex and targeted therapeutic approach. The CS backbone contributes inherent antibacterial and hemostatic properties, while light activation enhances ROS regulation, inflammation control, and vascular regeneration. For instance, CS–AuNP hydrogels under NIR irradiation exert localized photothermal antibacterial action while simultaneously stimulating angiogenesis and collagen deposition in diabetic wounds [165]. Similarly, CS methacryloyl (CSMA)-based hydrogels integrated with soy isoflavones and Au NPs demonstrated photo-triggered drug release, which leading to reduced inflammation and enhanced VEGF-mediated neovascularization in diabetic models [166].

An advanced CS-based multifunctional hydrogel was developed by Gao et al. [135] that combines carboxymethyl CS (CMCS), oxidized sodium alginate and gelatin with polydopamine-coated ZIF-8 nanoparticles (COG-Z@P200), forming a mild photothermal system for MRSA-infected diabetic wounds (Figure 6B). Upon NIR irradiation (808 nm), the composite generated localized heat (≈40–45 °C) and released Zn^2+^ ions, which synergistically disrupted bacterial membranes and metabolism achieving >99.5% MRSA elimination. Multi-omics analysis confirmed that the hydrogel suppressed glycolysis, the TCA cycle, and arginine biosynthesis while down-regulating quorum-sensing and virulence genes. In vivo, the system accelerated wound closure by 48%, promoted M2 macrophage polarization, enhanced VEGF-mediated angiogenesis, and increased collagen deposition without damaging healthy tissue. Further, the developed CS network provided biocompatibility, ROS scavenging, and pH-responsive degradation, making this photo-responsive CS hydrogel a potent, antibiotic-free platform for diabetic wound therapy through metabolic interference and microenvironment regulation. Overall, these demonstration highlight that photo-responsive CS hydrogels system allow precise, on-demand antibacterial and regenerative effects under light irradiation. They provide high control and strong antimicrobial activity but require external irradiation, limiting deep wound application.

### 6.4. Glucose-Responsive CS Hydrogel

Glucose-responsive CS hydrogels are smart biomaterials designed to sense elevated glucose concentrations in diabetic wounds and respond by triggering a therapeutic action [167]. Chronic hyperglycemia in diabetic wounds delays healing and contributes to inflammation, infection, and oxidative stress [168]. These hydrogels use the high-glucose microenvironment as a biological cue to regulate drug delivery, nitric oxide (NO) generation, or hydrogel degradation in a controlled way. Their responsiveness is typically achieved through three main mechanisms [169]: (i) the glucose oxidase reaction system, (ii) phenylboronic acid (PBA) modification, where PBA groups reversibly bind with glucose, altering the hydrogel structure and triggering swelling or release. (iii) the Concanavalin A (ConA)-mediated competitive binding system.

The glucose oxidase (GOx) catalyzes glucose to produce gluconic acid and hydrogen peroxide (H_2_O_2_). The products induce local chemical changes that promote drug release or hydrogel breakdown [170,171]. PBA and its derivatives are among the most widely used components for developing glucose-responsive hydrogels. Their responsiveness is based on the reversible covalent interaction between PBA and cis-diol groups. In a hyperglycemic environment, glucose molecules compete with the existing diol-containing components to form boronate ester bonds with PBA. This competitive binding weakens or disrupts the pre-formed PBA–diol crosslinks within the hydrogel network, leading to a decrease in crosslinking density. As a result, the hydrogel undergoes swelling, partial degradation, or a sol–gel transition, which can be strategically used to control the release rate of encapsulated therapeutic molecules and thus allowing on-demand glucose-responsive drug delivery [172,173,174]. ConA is a lectin protein capable of binding selectively and reversibly to carbohydrate molecules. Its glucose sensitivity arises from a competitive binding process, in which elevated glucose concentrations cause free glucose molecules to displace immobilized carbohydrate ligands attached to the hydrogel matrix. This displacement alters the noncovalent interactions between ConA and the bound ligands, leading to a reorganization of the hydrogel’s crosslinked structure. As a result, the network undergoes swelling or structural relaxation, which facilitates glucose-dependent and controlled release of therapeutic agents [19].

A representative system is the CAHG hydrogel, prepared by in situ crosslinking of L-arginine-coupled CS and GOx-modified hyaluronic acid based on Schiff-base reaction. In this system, GOx converts glucose into H_2_O_2_, which subsequently reacts with L-Arg to generate NO, a signaling molecule that promotes angiogenesis, collagen synthesis, and antibacterial defense (Figure 6C) [136]. This ensures therapeutic action only under high-glucose conditions, reducing unnecessary release in normal tissue. Glucose-responsive CS hydrogels offer self-regulated drug or NO release, closely mimicking biological feedback. They effectively address hyperglycemia-related stress but involve complex synthesis and enzyme instability.

### 6.5. ROS-Responsive CS Hydrogel

**Figure 7:**
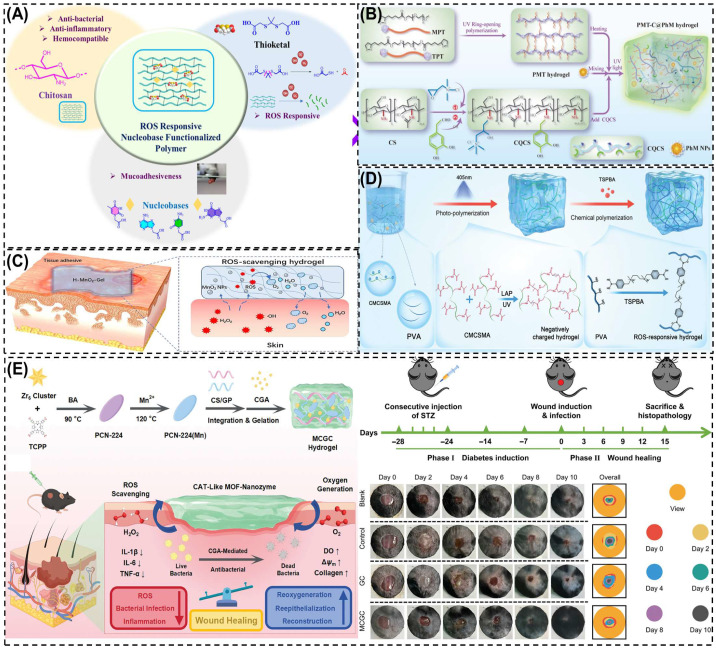
Schematic illustration of different ROS-responsive CS hydrogels for diabetic wound healing. (**A**) Nucleobase-functionalized CS hydrogel with thioketal linkers for ROS-triggered degradation and drug release. (**B**) Quaternized CS hydrogel (PMT-C@PhM) with MnO_2_ nanozymes mimicking SOD/CAT to remove ROS and enhance angiogenesis. (**C**) H-MnO_2_–CS hydrogel decomposing H_2_O_2_ into O_2_/H_2_O, reducing oxidative stress and inflammation. (**D**) Immunomodulatory CS hydrogel (NCRH) adsorbing cytokines, scavenging ROS, and promoting M2 macrophage polarization. (**E**) Dual-metal MOF-decorated CS hydrogel (MCGC) combining Mn/Zr nanozymes and CGA for ROS scavenging, oxygen generation, and accelerated wound repair. Reprinted with permission from Kulkarni et al. [175] (copyright, 2025, Elsevier), Wang et al. [176] (copyright, 2025, Elsevier), Wu et al. [177], (copyright, 2023, BMC), Wang et al. [178] (copyright, 2024, Wiley-VCH GmbH), and Wei et al. [137] (copyright, 2024, Wiley-VCH GmbH).

ROS-responsive CS hydrogels are designed to sense the abnormally high levels of ROS in diabetic wounds and convert that signal into therapy [169]. Two core strategies are used: (i) ROS-cleavable linkers such as thioketals and aryl boronic/boronate esters are grafted into CS networks. When H_2_O_2_ level rise, these linkers undergo bond scission, causing network loosening and enabling on-demand release of antibiotics, growth factors, or NO donors. For instance, Kulkarni et al. [175] designed a ROS-responsive nucleobase-conjugated CS hydrogel using thioketal linkers for oxidative stress-triggered drug release. The guanine-functionalized variant (P4) showed strong adhesion, ROS sensitivity, and antibacterial activity. Under H_2_O_2_ exposure, the hydrogel degraded to release drugs while scavenging ROS, reducing inflammation, and promoting tissue repair. As shown in Figure 7A, this system combines CS’s biocompatibility and antibacterial properties with ROS-cleavable bonds and nucleobase functionalization, enabling on-demand drug delivery and accelerated healing in diabetic wounds. (ii) ROS-scavenging components such as tannic/gallic acid, polydopamine, ceria/manganese dioxide (MnO_2_) nanozymes, Prussian blue, selenium and melanin-like nanoparticles are embedded in CS to catalytically quench ROS, and restoring redox balance. For example, Wang et al. [176] developed a ROS-responsive quaternized CS hydrogel incorporating thioctic acid-functionalized PEGs and polydopamine-coated MnO_2_ nanozymes to treat diabetic wounds (Figure 7B). The MnO_2_ nanozymes mimicked superoxide dismutase (SOD) and catalase activity, scavenging excess ROS and releasing oxygen to relieve hypoxia, while CS provided antibacterial and adhesive properties. The hydrogel promoted M2 macrophage polarization, angiogenesis, and collagen deposition, resulting in ~95% wound closure within 14 days. These combined effect make it an effective antioxidant and antimicrobial dressing for diabetic wound healing.

These hydrogels directly address diabetic-wound pathologies-persistent oxidative stress, infection, and delayed angiogenesis. By lowering excessive ROS, they limit tissue damage and macrophage M1 polarization, while ROS-triggered release supplies antimicrobials or pro-angiogenic signals exactly where needed. A ROS-responsive CS-based hydrogel (H-MnO_2_-Gel) was developed by incorporating hollow MnO_2_ nanoparticles (H-MnO_2_ NPs) into a quaternary CS–tannic acid matrix to alleviate oxidative stress and inflammation in chronic wounds [177]. The MnO_2_ nanozymes catalytically converted excess H_2_O_2_ into O_2_ and H_2_O, effectively scavenging ROS and restoring redox balance, while the CS network offered antibacterial activity, tissue adhesion, and biocompatibility (Figure 7C). The hydrogel protected fibroblasts from oxidative injury and reduced inflammatory cytokine expression, demonstrating strong potential for oxidative microenvironment regulation in diabetic wound healing. Another study by Wang et al. [178] developed an immunomodulatory CS-based negatively charged and ROS-responsive hydrogel (NCRH) capable of capturing pro-inflammatory cytokines and scavenging ROS to promote chronic diabetic wound remodeling. The hydrogel was synthesized from methacrylamide carboxymethyl chitosan (CMCSMA) and TSPBA-modified polyvinyl alcohol (PVA) via dual polymerization (Figure 7D). The negatively charged CMCSMA electrostatically adsorbed positively charged cytokines (IL-1β, TNF-α), while the ROS-responsive PVA network eliminated excess ROS. In diabetic models, NCRH systems consistently show reduced inflammatory cytokines, increased M2 macrophages, enhanced HIF-1α/VEGF signaling, faster re-epithelialization, and denser collagen I/III, culminating in accelerated closure. For intense, Wei et al. [137] developed a MOF nanozymes/chlorogenic acid/genipin crosslinked CS-based hydrogel (MCGC) with potent ROS-scavenging, oxygen-generating, and antibacterial properties for the treatment of infected diabetic wounds. As shown in Figure 7E, the hydrogel was synthesized by integrating Mn/Zr-based PCN-224(Mn) MOF nanozymes, chlorogenic acid (CGA), and a CS-gelatin (CS/GP) matrix via genipin crosslinking. Mechanistically, the Mn–Zr MOF nanozymes acted as catalase-like (CAT) mimics, catalytically decomposing excess H_2_O_2_ into oxygen, which reduced oxidative stress and relieved tissue hypoxia. In vivo diabetic wound studies revealed that the MCGC-treated group achieved the fastest wound closure, surpassing control and blank groups by Day 8, with evident re-epithelialization and collagen remodeling. The hydrogel not only eliminated ROS but also promoted cell proliferation (TNF-α/IL-6/ IL-1β), collagen synthesis, and angiogenesis, leading to accelerated wound healing. These results demonstrate that ROS-responsive CS hydrogels convert excessive oxidative stress, restore redox balance, and promote angiogenesis. They show superior antioxidative and healing performance but they rely on uncertain biodegradability. Developing natural antioxidant alternatives may improve clinical viability.

### 6.6. Dual-Responsive CS Hydrogel

Dual-responsive CS hydrogels are multifunctional materials designed to react to two biological stimuli simultaneously, such as pH/ROS, pH/glucose, or ROS/glucose. This dual action enables control over drug delivery and wound repair [179]. A common design involves pH/ROS-responsive CS-based hydrogels, which contain dynamic bonds like Schiff base and boronate ester linkages. When exposed to acidic and oxidative conditions of diabetic wounds, these linkages break, leading to controlled hydrogel degradation and site-specific release of therapeutic molecules such as antibacterial or angiogenic agents. Another approach combines ROS and glucose sensitivity, where thioketal or boronate groups are integrated with GOx to trigger drug or NO release only under both oxidative and hyperglycemic conditions. This dual response helps restore redox balance, enhances vascular growth, and reduces inflammation [180,181]. For instance, Li et al. [138] created an ROS/glucose-responsive quaternized CS hydrogel containing salvianolic acid B, which regulated oxidative stress and glucose metabolism to promote collagen formation and rapid wound closure.

Similarly, Dai et al. [182] designed a multi-stimuli CS-based hydrogel that responded to pH, ROS, and mild heat, improving angiogenesis and immune modulation in diabetic wounds. Overall, dual-responsive CS hydrogels can adapt to the changing wound microenvironment and deliver drugs when needed. They also maintain moisture balance, control inflammation, and stabilize oxidative conditions, making them a powerful platform for treating chronic diabetic wounds. However, their complex fabrication and batch-to-batch variability remain significant challenges. In addition, mechanical and stability limitations associated with added responsive components may affect long-term performance and clinical translation. Examples of some single and dual-stimuli-responsive CS-based hydrogel and wound healing mechanisms are summarized in Table 3.

## 7. Clinical and Commercial Translation of CS-Based Hydrogels

Although many studies on CS hydrogel have been conducted in animal models, several CS-based wound dressings have advanced to human clinical trials and commercial use. These developments demonstrate their strong translation potential. Products such as HemConTM, ChitoHeal, Celox^®^, and Kytocel^®^ have shown significant improvements in wound closure, infection control, and hemostasis in both hospital and field settings. These dressings highlight CS’s clinical versatility, combining biocompatibility, antimicrobial action, and moisture retention capacity. Table 4 summarizes the representative CS-based wound dressing materials evaluated at the human level and their therapeutic outcomes.

## 8. Research Gaps and Future Outlook

This review has identifies several significant gaps and future directions in bio-inspired CS-based hydrogel research. These findings can assist researchers in recognizing underdeveloped areas and determine, which aspects should be prioritized to overcome existing limitations. The main considerations are summarized below.

### 8.1. Safety Considerations Related to Hydrogel Degradation

One important aspect in the clinical use of CS-based hydrogels is the fate of incorporated metallic nanoparticle, small molecules, and synthetic crosslinkers after hydrogel degradation. As the polymer network gradually breaks down, these components may be released into the wound environment. If their concentration exceeds physiological tolerance, they can potentially cause cytotoxicity, oxidative stress or inflammatory response. For example, excessive release of silver or copper nanoparticles can impair fibroblast proliferation, while residual aldehyde or epoxide based chemical crosslinkers may delay epithelialization.

Recent studies address these concerns by using biocompatible or bioresorbable metals, natural crosslinkers such as Genipin or tannic acid and surface-modified nanoparticles with controlled-release coatings to minimize burst release. Future hydrogel designs should include careful evaluation of degradation products, long-term cytotoxicity, and in vivo clearance pathways to ensure both therapeutic efficacy and clinical safety.

### 8.2. Development of Clinically Reliable and Multifunctional Hydrogels

Achieving consistent clinical outcomes with CS hydrogels requires addressing variability in physicochemical properties arising from differences in polymer source, DD, and molecular weight. Such inconsistencies often result in variable performance during large-scale production. Establishing standardized fabrication protocols and robust quality control measures are essential to ensure reproducibility, scalability, and regulatory acceptance.

Future directions should focus on developing standardized fabrication protocols, implementing quantifiable control metrics (e.g., fixed DD/molecular weight ranges, impurity thresholds, gel strength standards), and validating this procedure under Good Manufacturing Practice (GMP)-compatible conditions to enable reproducible, scalable, and regulatory-ready CS hydrogel production.

### 8.3. Incorporation of Bioactive Molecules and Nanostructures

Integrating biologically active agents into CS-based matrices can further amplify their regenerative capacity. Growth factors, antimicrobial peptides, nitric oxide donors, and angiogenic proteins (such as VEGF or FGF) can be encapsulated within the polymeric network to accelerate vascularization and tissue remodeling. However, these compounds often suffer from instability or denaturation in hostile wound environments. To overcome this limitation, nanocarrier-assisted encapsulation using liposomes, polymeric nanoparticles, or metal–organic frameworks can provide sustained release and molecular protection.

Nanocomposite CS hydrogels embedded with metallic or oxide nanoparticles have shown notable antibacterial, antioxidant, and pro-angiogenic performance. Advancing, hybrid nano-bio systems that combine catalytic nanomaterials with CS scaffolds could be designed to regulate intracellular pathways such as macrophage polarization, collagen synthesis, and fibroblast activation. In addition, gene-responsive and immunomodulatory hydrogels that deliver nucleic acids (e.g., siRNA, miRNA, or CRISPR components) hold potential for directly influencing cell behavior and restoring normal wound physiology.

### 8.4. Advanced Manufacturing and Smart Design

Emerging fabrication approaches such as 3D bioprinting, electrospinning, and microfluidic templating offer precise control over material architecture, enabling the fabrication of wound dressings tailored to specific wound geometries. Three-dimensional bioprinting can produce patient-specific scaffolds with spatial gradients of drugs, growth factors, or responsive domains. Incorporating biosensors within these printed matrices could transform CS hydrogels into theragnostic systems, capable of monitoring wound conditions such as pH, oxygen, and glucose while simultaneously delivering therapeutics.

Injectable and self-healing hydrogels also represent an important focus for future development. Hydrogels with dynamic covalent networks, such as those containing Schiff-base or boronate ester linkages can repair structural damage autonomously and extend dressing lifespan. Thermosensitive injectable hydrogels, which solidify at body temperature are particularly advantageous for treating deep or irregular wounds. Additionally, coupling conductive or magnetically responsive materials with CS networks may allow electro stimulated or remotely triggered wound repair, enhancing healing precision and comfort.

### 8.5. Computational and Artificial Intelligence Approaches

Artificial intelligence (AI) and computational modeling can accelerate the optimization of CS hydrogel formulations. Machine learning can predict key relationships among polymer composition, crosslinking density, degradation kinetics, and biological performance, guiding rational material design while reducing experimental workload. Furthermore, AI-assisted wound monitoring through digital imaging and pattern recognition could provide real-time insights into healing progression and hydrogel functionality. Integrating these data-driven strategies with experimental validation will support a new generation of predictive biomaterial engineering.

### 8.6. Translational Pathways and Preclinical Assessment

Although laboratory studies demonstrate significant potential, clinical implementation remains limited by insufficient long-term and large-animal evaluations. Current research predominantly relies on rodent models, which cannot fully reproduce the complexity of chronic diabetic wounds in humans. Future investigations should adopt clinically relevant preclinical models, standardized evaluation parameters, and extended follow-up durations to assess safety, immunogenicity, and degradation behavior comprehensively.

Regulatory approval also presents a significant hurdle, particularly for composite materials incorporating biological or nanostructured components. Close collaboration between researchers, clinicians, industry partners, and policymakers is needed to establish uniform testing standards and GMP–compliant protocols. Ensuring scalability while maintaining bioactivity and mechanical integrity will be crucial for industrial translation.

### 8.7. Future Vision for Next-Generation CS Hydrogels

Over the next decade, stimuli-responsive CS hydrogels are expected to advance significantly. They will likely evolve into bio-intelligent and multifunctional wound dressings capable of autonomous therapeutic action. These systems will combine multiple functionalities-antimicrobial, anti-inflammatory, antioxidative, and pro-angiogenic within a single, environmentally adaptive framework. The concept of living hydrogels-incorporating engineered or probiotic cells capable of secreting healing-promoting molecules offers an exciting new direction in regenerative therapy.

Sustainability will also become a key focus with emphasis on eco-friendly synthesis routes, renewable CS sources, and biomimetic crosslinking methods that reduce environmental impact and cost. Beyond diabetic wound treatment, such adaptive CS-based materials have potential applications in surgical healing, soft tissue engineering, and targeted drug delivery. Ultimately, success in this field will depend on synergistic collaboration among multidisciplinary scientists to translate laboratory innovations into practical and patient-centered therapies.

## 9. Conclusions

Stimuli-responsive CS hydrogels have emerged as advanced biomaterials for diabetic wound management. They combine the intrinsic biocompatibility, biodegradability, and antimicrobial nature of CS with responsiveness to physiological signals such as pH, glucose, temperature, light, and ROS. By dynamically interacting with the wound microenvironment, these hydrogels can modulate inflammation, suppress microbial proliferation, promote angiogenesis, and accelerate tissue regeneration. Their capability to provide localized and controlled therapeutic release allows precise regulation of the healing process, establishing them as a promising alternative to conventional wound dressings. However, challenges related to mechanical durability, batch-to-batch reproducibility, regulatory translation, and sterilization compatibility still limit large-scale clinical adoption. The progressive evolution of CS hydrogels toward intelligent, multi-stimuli-responsive systems marks a significant advancement in wound-healing technology. Continued innovation in material design, functional modification, and regulatory alignment is expected to facilitate their transition from laboratory studies to practical medical use.

## Figures and Tables

**Table 3:** Examples of single and dual-stimuli responsive CS-based hydrogels and their therapeutic applications in diabetic wound healing.

Type of Stimuli	Stimulus	Hydrogel Composition	Therapeutic Application in Diabetic Wound Healing	Reference
Single	pH	N-carboxyethyl CS (N-CS) crosslinked in situ with adipic acid dihydrazide (ADH) and hyaluronic acid–aldehyde (HA–ALD)	Sustained insulin release for diabetic wound healing	[147]
Single	Temperature	CS hydrogel incorporating copper–magnesium bimetallic metal–organic framework (Cu/Mg-MOF) nano-enzymes	Modulates wound microenvironment to enhance diabetic wound healing	[134]
Single	Photo	Carboxymethyl CS (CMCS), gelatin, and oxidized sodium alginate combined with polydopamine-coated ZIF-8 nanoparticles	Under NIR irradiation, promotes M2 macrophage polarization, enhances angiogenesis, and stimulates collagen deposition	[135]
Single	Glucose	CS–hyaluronic acid–L-arginine (CAHG) hydrogel	Promotes angiogenesis, collagen synthesis, and antibacterial defense in diabetic wounds	[136]
Single	ROS	CS hydrogel (PMT-C@PhM) with polydopamine-coated MnO_2_ nanozymes	Induces M2 macrophage polarization and enhances collagen regeneration	[176]
Dual	pH and glucose	Injectable carboxymethyl CS–hyaluronic acid hydrogel mimicking the ECM	Effective for diabetic chronic wound healing, and skin regeneration	[180]
Dual	Glucose and ROS	Hyaluronic acid–glycol CS hydrogel grafted with phenylboronic acid and catechol side groups	Accelerates healing via anti-inflammatory activity, ROS scavenging, and improved tissue adhesion	[139]

**Table 4:** Commercially available CS-based hydrogel dressings for wound healing applications [183].

Product	Material	Applications	Producer
ChitoHeal	N-Acetylglucosamine	Wound dressings for skin tissue regeneration	ChitoTech
Chitopack C^®^	CS acetate salt, ethylene glycol, ice, and NaOH		Eisai
Wellife^®^ LB-01	CS		Wellife Medical
Tegasorb^®^	CS particle		3M
TraumaStat^®^	Freeze-dried CS containing highly porous silica		Ore-Medix
KytoCel	CS	Wound dressings for absorbing wound exudate	Aspen Medical
Chitoderm plus^®^	Strong superabsorber coated with chitosan		Trusetal
HemConTM	Freeze-dried chitosan acetate salt	Hemostatic dressings	Tricol Biomedical
Axiostat^®^	CS		Axiobio
ExcelArrestTM	Modified CS		Hemostasis
ChitoClot Pad	CS		BenQ Materials BioMedical
Chitoclear^®^	Chitoclear^®^ positively charged CS		Primex
Celox^®^	Chito-R^®^ activated CS		MedTrade

## Data Availability

Data are available within the manuscript.
